# Using Social Media Data to Investigate Public Perceptions of Cannabis as a Medicine: Narrative Review

**DOI:** 10.2196/36667

**Published:** 2023-02-27

**Authors:** Sedigh Khademi, Christine Mary Hallinan, Mike Conway, Yvonne Bonomo

**Affiliations:** 1 Department of General Practice Faculty of Medicine, Dentistry & Health Sciences University of Melbourne Victoria Australia; 2 Centre for Health Analytics Murdoch Children's Research Institute Melbourne Australia; 3 Health & Biomedical Research Information Technology Unit Faculty of Medicine, Dentistry and Health Sciences University of Melbourne Melbourne Australia; 4 School of Computing and Information Systems University of Melbourne Melbourne Australia; 5 Department of Addiction Medicine St Vincent's Health Melbourne Australia

**Keywords:** social media, medicinal cannabis, public health surveillance, internet, medical marijuana

## Abstract

**Background:**

The use and acceptance of medicinal cannabis is on the rise across the globe. To support the interests of public health, evidence relating to its use, effects, and safety is required to match this community demand. Web-based user-generated data are often used by researchers and public health organizations for the investigation of consumer perceptions, market forces, population behaviors, and for pharmacoepidemiology.

**Objective:**

In this review, we aimed to summarize the findings of studies that have used user-generated text as a data source to study medicinal cannabis or the use of cannabis as medicine. Our objectives were to categorize the insights provided by social media research on cannabis as medicine and describe the role of social media for consumers using medicinal cannabis.

**Methods:**

The inclusion criteria for this review were primary research studies and reviews that reported on the analysis of web-based user-generated content on cannabis as medicine. The MEDLINE, Scopus, Web of Science, and Embase databases were searched from January 1974 to April 2022.

**Results:**

We examined 42 studies published in English and found that consumers value their ability to exchange experiences on the web and tend to rely on web-based information sources. Cannabis discussions have portrayed the substance as a safe and natural medicine to help with many health conditions including cancer, sleep disorders, chronic pain, opioid use disorders, headaches, asthma, bowel disease, anxiety, depression, and posttraumatic stress disorder. These discussions provide a rich resource for researchers to investigate medicinal cannabis–related consumer sentiment and experiences, including the opportunity to monitor cannabis effects and adverse events, given the anecdotal and often biased nature of the information is properly accounted for.

**Conclusions:**

The extensive web-based presence of the cannabis industry coupled with the conversational nature of social media discourse results in rich but potentially biased information that is often not well-supported by scientific evidence. This review summarizes what social media is saying about the medicinal use of cannabis and discusses the challenges faced by health governance agencies and professionals to make use of web-based resources to both learn from medicinal cannabis users and provide factual, timely, and reliable evidence-based health information to consumers.

## Introduction

### Cannabis as Medicine

Public interest in the use of cannabis for medical purposes is rising globally, which has resulted in some countries enacting legislation, leading to increased access to cannabis and cannabis-based products. These legislative changes have generated public health concerns, as evidence for the efficacy and safety of cannabis is yet to be fully established, while cannabis products are increasingly being provided to consumers by a cannabis industry using sophisticated marketing strategies to influence public understanding of its benefits [1].

There has been growing scientific research into the therapeutic benefits of cannabis plant–derived cannabinoids, which has helped fuel consumer and industry interest in cannabis as medicine. The scientific literature shows that cannabinoids possess many pharmacological effects including anticonvulsant, neuroprotective, analgesic, anxiolytic, antidepressant and antipsychotic, antioxidant, anti-inflammatory, and immunomodulatory properties [2]. Medicinal cannabis is used to treat chronic and cancer-related pain, depression, anxiety, sleep disturbance, and neurological disorders such as epilepsy and multiple sclerosis [3].

Research along with increased consumer use of medicinal cannabis and other cannabis products has contributed to a reassessment of the negative perception of cannabis, which has prevailed since its worldwide prohibition (eg, the US Federal Government’s Marihuana Tax Act of 1937 [4]). However, the acceptance and use of cannabis as medicine has run ahead of research evidence, and it has been difficult for consumer law to keep pace [5,6]. The traditional requirements of drug trials, approvals, and postlicensure surveillance are either not applied or not consistently applied to medicinal cannabis. Consequently, there is a public health concern that evidence for the efficacy and safety of medicinal cannabis is not fully established for a range of clinical contexts [7]. The need for more knowledge about the efficacy, side effects, dosage, and use of cannabis products has led to an interest in collecting information about medicinal cannabis use and effects from many available sources, an important one being social media [6].

### Social Media as a Data Source

Social media, health forum conversations, and web-based queries provide a rich resource for understanding and studying health-related information. This web-based, user-generated text can be used to investigate self-reporting of a wide range of health-related conditions, communicable and noncommunicable diseases, substance use (both legal and illegal), mental health issues, and the impact of policy on health outcomes [8].

This narrative review [9] examines what social media research on the use of cannabis as medicine reveals, providing a scholarly summary of the key findings of recent studies based on user-generated social media text as a data source. The studies used for the review came from our previous systematic scoping review of papers that researched social media discourse on cannabis as medicine [10]. Our previous review [10] examined how user-generated texts are being used for medicinal cannabis research.

This review summarizes the findings of those studies, grouping them according to their most common themes, which describe the perceived benefits of medicinal cannabis. The review then discusses the relevance of the information for consumers and researchers and draws conclusions applicable for health professionals and health organizations. To our knowledge, this is the first review that summarizes and synthesizes the findings of studies that use web-based user-generated data sources for medicinal cannabis research.

In this paper, we set out to answer these research questions:

- What does research into web-based user-generated text reveal about the use of cannabis as medicine?
- What does social media reveal about public perceptions of and sentiments toward cannabis?
- How are consumers influenced by social media interactions regarding cannabis as medicine?

## Methods

**Figure 1:**
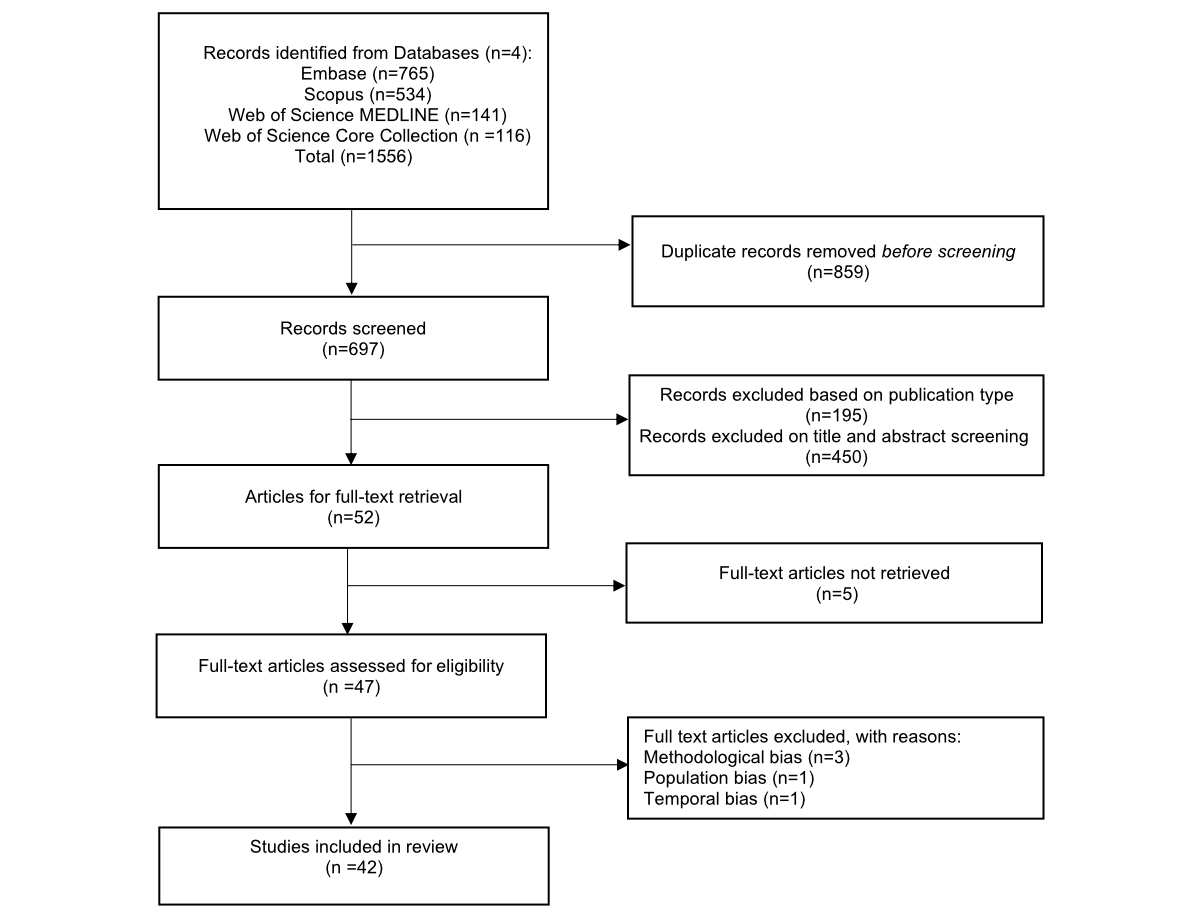
The PRISMA (Preferred Reporting Items for Systematic Reviews and Meta-Analyses) diagram.

This paper is based on studies that were previously selected for our systematic scoping review of social media discourse on cannabis as medicine. We searched for English language studies published between January 1974 and April 2022 and indexed in the MEDLINE, Embase, Web of Science, and Scopus databases. The search terms included social media terms, or search engine terms and health- and medical-related terms. The details of the search terms are presented in Multimedia Appendix 1 (Table S1). The studies were included if web-based user-generated text was used as a data source for research into the use of cannabis for medicinal purposes or as a data source for health-related studies with findings related to medicinal cannabis (Multimedia Appendix 1, Table S2). The list of articles included in this paper is presented in (Multimedia Appendix 2 [11-52]). A PRISMA (Preferred Reporting Items for Systematic Reviews and Meta-Analyses) flowchart is shown in Figure 1.

The scoping review focused on the research rigor and research motivations of the papers, and a separate technical review provided an overview of the techniques, limitations, and challenges faced by these studies [53]. This narrative review presents a summary of the findings of those studies to draw conclusions about the social media portrayal of cannabis as medicine, which predominantly speaks of its benefits, and to observe the use and influence of social media on consumers’ perceptions of medicinal cannabis. The results are structured around themes discovered in the findings of the studies.

## Results

### Overview

A content analysis of the findings of the included studies revealed 2 major themes. One theme related to benefits (and potential risks) of medicinal use of cannabis, which included reports of physical and mental health benefits of cannabis and that cannabis is also seen as an important therapeutic for severe conditions including pain and terminal conditions such as cancer. There were also papers related to the use of cannabidiol (CBD), adverse events, and sentiments toward cannabis. The other major theme related to the use, influence, and quality of web-based information, and information pathways around cannabis.

### Use of Cannabis as Medicine

Discussions on the benefits of cannabis as medicine were overwhelmingly prevalent, balanced in part by descriptions of the risks of cannabis consumption for medical reasons.

#### Cannabis-Related Studies

In this review, 21% (9/42) of the studies discussed general cannabis-related conversations regarding subjects such as legislation, the cannabis industry, and consumption patterns or social media–related issues such as social bots (which are automated accounts that manipulate social media discourse [54]). They were included in this review, as they discovered conversations containing mentions of the physical and mental health benefits of cannabis consumption.

In a corpus of cannabis-related tweets, it was found that health and medical discussions were the third most prevalent theme in nonbot conversations, where cannabis was recommended for a disparate range of conditions including cancer, plantar fasciitis, Crohn disease, sleep, pain, anxiety, depression, trauma, and posttraumatic stress disorder (PTSD) [11].

A related study mapped mentions of health effects and consequences in nonbot cannabis-related tweets to a medical dictionary and found that medical terms could be derived from the conversations. These posts discussed mental health, pain, and stress in the immune system among other health effects [12].

Health-related discussions were a repeating theme in approximately 1.2 million cannabis-related American and Canadian geotagged tweets [13] and in a further 40,000 American tweets [14]. The health-related theme also appeared in a qualitative analysis of 7000 tweets [15] from highly influential Twitter users (users who were in the top 25th percentile for number of followers and influence, measured by the Klout score).

Dabbing refers to the heating of cannabis concentrate and passing the vapor through a water pipe before inhalation [16]. A study of 3540 dabbing-related tweets found the health benefits of dabbing to be the fifth most popular theme, with claims of dabbing improving sleep, relieving anxiety, and assisting in problem-solving [17]. The study also described extreme negative effects of dabbing such as adverse respiratory effects and loss of consciousness. Edibles are cannabis-infused drinks or food such as cookies, teas, and *gummies* [55]. A study on cannabis edibles on Twitter found the facilitation of sleep as one of the most frequently reported benefits of edibles [18].

An analysis [19] of web-based cannabis product reviews on YouTube found that videos often shared personal and positive experiences of using cannabis. These experiences included euphoria; improvements in physical and mental health such as relaxation, pain relief, and sleep quality; and increased stamina. A qualitative content analysis of over 40,000 cannabis-related tweets found that cannabis was characterized as being much less harmful than conventional drugs for a variety of conditions such as sleep disorders, generalized anxiety, depression, and cancer. Less than 1% of the tweets indicated any concern about cannabis use, and 43% of them were anecdotes and personal stories [20].

#### Health-Related Studies

##### Overview

Of the studies, 62% (26/42) were either concerned with health problems and found descriptions of the benefits of cannabis (including CBD) or were primarily interested in the use of cannabis for a specific health condition.

An examination of pregnancy- and cannabis-related posts on Twitter found 3 major themes. These were how to safely continue cannabis use during pregnancy and the postpartum period and how to manage pregnancy-related symptoms such as morning sickness, nausea, vomiting, headaches, pain, stress, and fatigue [21].

Self-medication for conditions such as PTSD, anxiety, sleep disorders, and pain was the topic in over a third of 974 cannabis-related Reddit posts in the Veterans subreddit [22]. A study of PTSD-related tweets found that 5% of them were cannabis-related and 90% of those posts were supportive of cannabis use for PTSD [23].

An analysis of cancer-related queries in Google Trends from 2011 to 2018 [24] found that the volume of cannabis-related cancer queries doubled during the study period; however, during the same time frame, the number of searches for standard cancer therapies increased only marginally. The study used the BuzzSumo social media analyzer, which evaluates social media users’ engagement with news stories, and found that 50% of high-impact news were anecdotes of patients with cancer cured by cannabis. However, an analysis of 13 years of conversations from the Cancer Survivor Network found that cannabis was primarily used for nausea [25].

An analysis of 2 subreddits, one related to opioid use and the other related to opioid recovery, found that although cannabis-related posts were not common, they were twice as prevalent in recovery conversations versus use conversations. Cannabis was also mentioned for the management of opioid withdrawal symptoms including anxiety, insomnia, and pain [26].

##### Cannabis as Complementary and Alternative Medicine

There is increasing interest in complementary and alternative medicine (CAM), inspired by desires for healthier lifestyles, dissatisfaction with conventional therapies and the adverse effects of allopathic medicines, and an aversion to the omnipresence of the pharmaceutical industry [56]. Studies in this section found that cannabis was mentioned as complementary medicine for a variety of health conditions such as glaucoma, asthma, and bowel disease.

A thematic analysis of glaucoma-related tweets found that where complementary therapy was discussed, 87% highlighted the use of medical cannabis as a treatment option [27]. A content analysis of asthma-related videos showed that the most common theme was complementary medicine, and cannabis was mentioned as an option for asthma treatment [28].

An analysis of tweets from the bowel disease community found that medicinal cannabis was the fourth most frequently mentioned term [29] and that patients were actively engaged in discussing the medical benefits of cannabis to alleviate common symptoms of bowel disease, specifically diarrhea, constipation, and pain. Medicinal cannabis was the most frequently mentioned therapeutic, and overall, it was discussed positively.

Data from crowdfunding campaigns on GoFundMe [57] were used to explore the reasons of patients with cancer for using cannabis as CAM [30]. The study’s findings showed that CAM users were more likely to be in an advanced stage of cancer. They found that the three main reasons for using CAM were (1) as an adjunct to conventional cancer therapies for curative and therapeutic purposes; (2) to alleviate both cancer symptoms and side effects of conventional therapies including pain, stress, and anxiety; and (3) dissatisfaction with other medical treatment options. There were a considerable proportion of CAM users (34.8%) who chose to forgo standard therapies, reporting that they were either too toxic for the body or that their cancer was too advanced. CBD was found to be among the top 10 most commonly used alternative treatments.

##### Cannabis for Pain

Cannabis was reported as an effective pain management alternative to opioids [31]. In a collection of tweets from individuals who self-identified as addicted to opioids, it was found that cannabis use featured in topics about taking illicit drugs in recovery from dependence and for pain management [32].

Examination of Facebook support group posts on brachial plexus injury identified alternative treatments as an emerging theme for pain management and that many posts promoted cannabinoids as a safe and preferred alternative for opioid-based pain medication [33]. An analysis of pain-related tweets in Ireland found cannabis to be the fourth most occurring keyword [34]. However, although approximately 90% of cannabis-related tweets were highly positive, they were mostly nonpersonal (they did not report on the experience of an individual) and claimed to be supported by medical research—designed to generate awareness and offer advice on the benefits of cannabis.

##### Cannabis as a Last Resort Therapeutic

Cannabis is featured in discussions regarding last-resort therapeutics, especially when a condition was treatment resistant or where no viable treatment options were available. In a thematic analysis of alternative medicine forum conversations regarding cluster headaches and migraines, it was found that those affected by headaches pursued psychoactive substances as a last-resort treatment [35]. Cannabis was frequently mentioned for its potential to mitigate symptoms or to decrease the incidence of migraine attacks. Some had used cannabis for other reasons but coincidentally found that it helped headaches. Others indicated that higher doses of cannabis can trigger headaches. There were mentions of the timing, mode of use, and dosage influencing the benefit or lack thereof of cannabis. The study found that cannabis-related conversations contained more contradictory information than posts on other alternative medicines.

Autism spectrum disorder is a condition that has significantly increased in prevalence in the last decade [58,59]. As with many other developmental disorders, there is considerable uncertainty regarding its etiology, and there are many questions regarding appropriate treatments [60]. An analysis of Google Trends queries on the causes and treatments of autism spectrum disorder showed that cannabis was the second most-searched term after applied behavior analysis and that cannabis-related searches showed an increasing trend [36].

Attention-deficit/hyperactivity disorder (ADHD) is considered as a risk factor for cannabis use disorder [61,62]. A study that analyzed web-based forum discussions about the effect of cannabis on ADHD found that 63% of the posts claimed a therapeutic effect by improving symptoms of inattention or hyperactivity-impulsivity. However, that 20% of the posts indicated that cannabis worsened ADHD symptoms and that 12% of the discussions indicated both therapeutic and adverse effects. A further 5% of posts commented that cannabis had no effect on ADHD symptoms [37].

##### The Focus on CBD

CBD is a cannabinoid derived from cannabis with a high potential for medicinal use [63]. One of the studies analyzed search volumes for CBD on Google Trends and showed that there was a substantial increase in CBD searches since 2014, with a 6.4-million growth during April 2019 [38]. CBD was the fourth most-searched alternative medicine compared with other health topics, products, or alternative medicines. A later study [39] assessed a random sample of 3000 Reddit posts for the use of CBD for conditions where evidence-based therapies currently exist. Of these, 376 were identified as personal testimonies, with 90% of them describing therapeutic effects from taking CBD. Psychiatric conditions were most frequently mentioned in the posts, followed by orthopedic, sleep, neurological, gastroenterological, addiction, cardiological, dermatologic, ophthalmologic, oral health, and sexual health conditions.

An examination of Reddit posts related to perceived therapeutic effects and popular modes of CBD use [40] showed anxiety disorder and pain as the 2 main conditions benefiting from CBD, with mentions of treatment for inflammation, headaches, sleep disorder, seizure disorders, nausea, and cancer. The most popular CBD products were oil and tinctures ahead of vapes, edibles, pills, and topicals. Vaping is the practice of inhaling cannabis vapor from an electronic device [64]. An analysis of vaping-related conversations found health discussions specifically on vape-administered CBD as a treatment for COVID-19 [41].

An analysis of CBD-related posts on Pinterest found that CBD was touted for mental and physiological benefits including pain relief, anti-inflammation, chronic disease treatment, and sleep improvement [42].

Another study examined CBD tweets and found that almost half of the CBD tweets are marketing related, where CBD is claimed to have therapeutic effects for pain, anxiety disorders, sleep disorders, and stress [43].

A study of CBD-related tweets compared its mentions in marketing messages with those in personal messages and found that there was an alignment between the medical conditions mentioned in both categories. However, where autism was discussed, they found that although marketing messages were positive in promoting CBD as a treatment, the overall messaging and sentiment in personal posts was negative, with many questioning the effectiveness of CBD for autism [44].

A study of CBD-related crowdfunding campaigns revealed that campaigners were looking for help to purchase CBD for cancer, seizure-inducing diseases and conditions, joint and inflammatory diseases, mental health disorders, nervous system diseases, and autoimmune diseases [45].

Another study [46] examined CBD-related crowdfunding campaigns to understand how CBD is represented as cancer-related care and showed that CBD is used for therapeutic and life-prolonging purposes and is presented as an effective treatment option. Patients with later-stage cancer were found to exclusively use CBD, incorporate it into alternative treatment plans for curative purposes, or extend life expectancy.

#### Adverse Effects of Cannabis

Although the studies revealed that discussions were mostly very positive toward cannabis as medicine, some of them noted negative consequences of cannabis use, and 1 of the studies focused on the detection of potential adverse effects of cannabis consumption [47]. This US-based study showed a high correlation between the side effects recorded on established reporting systems such as the Food and Drug Administration’s Adverse Drug Reporting System (FAERS) and those found in search engine queries. These included common side effects of anxiety, depression-related symptoms, psychotic symptoms such as paranoia and hallucinations, coughing, and other symptoms. The FAERS recorded more acute side effects of cannabis such as panic, hyperhidrosis, asthenia, and pyrexia, whereas Bing search logs detected more common side effects such as pain, headaches, and seizures, which were underreported in the FAERS.

A study of approximately 100,000 edibility-related cannabis tweets found that the overall opinion toward edibles was positive, being perceived as a safe mode of cannabis use. Negative tweets disclosed adverse effects that were largely attributed to the unreliability of the content details on the packaging of the edibles (ie, percentage of tetrahydrocannabinol or percentage of CBD) or the consumer’s lack of knowledge concerning edibles (ie, effects around excess consumption) [18].

A quantitative analysis of 400,000 Reddit discussions about smoking, vaping, edibles, dabbing, and butane hash oil concentrate use found fewer adverse event mentions related to new modes of use. The most frequently mentioned adverse effects were anxiety-related, linked to smoking, edibles, and butane hash oil, and “cough” mentions related to vaping and dabbing [48]. A continuation of the study further analyzed dabbing-related questions and found that questions about respiratory effects, anxiety, and vomiting were major components in a theme about health concerns [16]. An examination of 116 dabbing-related YouTube videos, of which 22% mentioned self-medication, found that only 21% of videos included some sort of warning around the potential for injury and negative side effects [49].

A study of cannabis self-help–related forums found that individuals actively sought help for cannabis use and addiction issues, including negative symptoms and intoxication or withdrawal issues [50]. A study on the unwelcome consequences of excessive dabbing reported adverse respiratory effects and intense experiences such as loss of body control, vomiting, and loss of consciousness [17]. Mapping Twitter conversations to a consumer-oriented medical dictionary found mentions of cannabis consumption health consequences, including impacts on the respiratory system, stress to the immune system, and gastrointestinal problems [12].

### Sentiments About Cannabis

Evaluations of social media sentiments toward cannabis and its products were performed in 19% (8/42) of studies, where they all found that social media posts were predominantly positive [15,18,20,23,29,34,44].

In total, 14% (6/42) of studies evaluated the impact of state policy on cannabis-related tweet sentiments and volume. These studies showed that more restrictive policies (where cannabis is either illegal or only legal for medical use) were associated with increased negative sentiments and less volume of social media posts compared with environments with less-restrictive policies where nonmedical use of cannabis is legal [13,17-19,23,51]. However, it is possible that an increased interest in cannabis for both medical and nonmedical use has resulted in legalization changes that reflect a community’s sentiments [24].

### The Use and Influence of Web-Based Information

Of the reviewed studies, 19% (8/42) examined the use of web-based information, including social media, by consumers when making choices about cannabis.

An information pathway describes the assimilation of important pieces of information in progressing along a path to reach a decision [65]. Data obtained from crowdfunding campaigns were used to identify the information pathways for deciding to use CBD for medical reasons. The major pathways were self-directed research followed by recommendations from a trusted care provider and shared experiences from other users in or associated with the individual’s personal network, which included social media messages and testimonials shared on the web [45].

A study on dabbing-related questions indicated that given the lack of evidence-based information regarding safe dabbing practices, people share personal experiences to learn how to dab and how to avoid its potential risks such as respiratory effects and anxiety. Personal experiences of active drug users of new and emerging substances were found to be the only source of information on alleged safe and effective use of these substances [16]. A mixed methods study of social media analysis and a survey of young adult cannabis users from the United States found that cannabis marketing had an influence on their decision-making. Approximately one-third of the participants had viewed cannabis product reviews in the prior month, and medicinal cannabis users were much more likely to view cannabis product reviews for information compared with nonmedical users [19].

A study of GoFundMe found that campaigners used anecdotal claims of CBD efficacy to support their funding requests and in doing so seldom referenced evidence-based sources of information [46].

These examples describe the use and influence of web-based information in the decision-making process. However, web-based information quality was variable and not always aligned with scientific evidence [66].

A study of the most shared news stories about cancer treatments described a story claiming that cannabis was a cancer cure that had attracted 4.26 million social media engagements but that the study was not based on any credible scientific evidence. In contrast, another article that the authors stated was accurate and that debunked the story attracted only 0.036 million engagements [24].

A study of glaucoma-related web-based posts reported that 2% of the annotated tweets had misleading information, mostly related to the medicinal use of cannabis for glaucoma [27].

An assessment of the content and quality of web-based information on cannabis and glaucoma from Google, Facebook, and YouTube found the information to be of varying quality [52], with 35% of reviewed websites and more than half of YouTube videos being presented by medical cannabis organization affiliates and lacking any professional medical authorship.

Social bots have been found to market unsubstantiated health claims in other domains [67] and to spread low-credibility information [68]. In a corpus of cannabis-related tweets, 14.5% of the posts were generated by bots, with health and medical claims being the second most prevalent subject [11].

## Discussion

### Overview

This narrative review summarizes the findings of studies using web-based user-generated text as a data source to study the use of cannabis as medicine. This paper provides insights into the indications for which medicinal use of cannabis is occurring and the role of web-based user-generated text for consumers and for research purposes.

### Indications for Cannabis Use

The studies found that cannabis was purported to help with a range of health conditions including cancer, sleep disorders, chronic pain, opioid use disorder, headaches, asthma, bowel disease, anxiety, depression, trauma, and PTSD, among other health issues. Cannabis was also described by some consumers as a boost to health, cited for its ability to help with relaxation and sleep, for enhancing stamina, and even to assist with creative activities and problem-solving [17]. The motivations for pursuing cannabis as alternative or complementary medicine described in the studies included failure of conventional treatments, their unwelcome side effects, and a desire to find more natural solutions, often in a context of uncontrolled or terminal disease and severe pain [30,45,46], or for addiction to prescribed or illicit drugs. This, coupled with the promotion of cannabis as natural and safe medicine, leads people to conclude that they have nothing to lose in trying cannabis.

Unclear or inaccurate labeling of cannabis products can lead to unexpected or adverse effects. Some studies found that less than 40% of CBD products were accurately labeled, while the rest were either underlabeled or overlabeled or did not state the presence of other ingredients such as the psychoactive tetrahydrocannabinol [69,70].

While cannabis is generally well tolerated, it has been reported to have a potential for negative effects, particularly among older adults and people with chronic disease, or owing to negative medication interactions [71]. There were cohorts in the study, such as pregnant women, who were long-term cannabis users and were concerned to know the health impacts of its continued use. This highlights that people who are susceptible to experiencing the ill effects of cannabis would benefit from accurate information and advice and monitoring by health providers.

### Social Media for Consumers

These studies found that social media and the internet are used by the public to seek and share information about the medicinal use of cannabis. It was found that both internet searches and the support of web-based communities had a strong influence in deciding to use CBD. People trying new formulations or experimenting with new modes of use found advice from other users rather than health professionals, and these sources were often found on the web. Previous studies have found that consumers more frequently use the internet as their first source of information and consider the collective wisdom found on social media as a credible and accessible source of information [72,73]. Other studies have indicated that the growth in web-based cannabis resources is often accompanied by a lack of balanced information such as promotional articles, which purport to be scientific. These resources have been shown to contain misleading information regarding the medical effects of cannabis [74,75]. Therefore, despite the convenience and support offered by social media and web-based forums, consumers should be aware that information on the web is largely unregulated and that health professionals need to be more proactive in obtaining and disseminating accurate information to help their clients reach informed decisions.

### Social Media as a Data Source for Research

The studies found web-based groups dedicated to discussions of both cannabis use and health problems and found that users mostly felt very comfortable discussing their concerns. Studies have shown that engagement with web-based support enables anonymous communication about stigmatized topics such as mental health [76], sexual abuse [77], and intimate matters [78], giving people a safe space to seek support and knowledge and share their concerns. People are drawn to social media and online support groups to manage stigma, gather information, and belong to a supportive and like-minded community. In the studies reviewed here, social media platforms provided researchers with insight into individuals’ intimately expressed struggles with health issues such as pain, addiction, and PTSD and the exploration of emerging drug-use practices such as dabbing and the use of edibles.

Despite the breadth of information that can be obtained from social media, researchers need to be aware of biases and inaccuracies in this data source as well as in data processing and analysis. The 2 main issues with data quality in social data are sparsity and “noise” [79]. One of the major contributors to noise in these papers were posts that promoted specific products, health claims, or ideas—these came either from the placement of advertising and promotional material by cannabis marketing companies or from social bots.

Identifying promotional posts and social bots should be one of the first steps in refining user-generated data for research purposes. Researchers need to be aware of the extensive presence of marketing companies and separate their posts from personal discussions, especially if the research aims to explore personal experiences around a subject. Attention should also be placed on defining a representative sample of the population of interest. Research questions and goals, data collection, and research methods must be defined with an understanding of the varied qualities of social media data sources.

### Limitations of This Study

Our literature search was limited to peer-reviewed publications in the English language indexed in the MEDLINE, Scopus, Web of Science, and Embase databases. The choice of databases and keywords for the literature search could have impacted the number of studies selected for this review. We identified biases in the data source and the analysis techniques of the studies included in the review, which could affect the generalizability of their results. Some of the data source biases are inherent to social media, such as demographics of people using social media and limited access to the data determined by platform policies [79]. For example, Twitter’s public application programming interface has historically only allowed access to 1% of tweets in the past, and the sampling techniques are unclear. Other biases that could have been accounted for by researchers were posts emanating from bots and nonindividual accounts, data selection owing to keyword choices [80], and validation of results by comparison with other data sources. Notwithstanding these limitations, this study has provided a comprehensive picture of research in this field, highlighting areas to be considered by consumers, researchers, health professionals, health organizations, and industry regulators.

### Conclusions

This narrative review helps to elucidate the major themes of medicinal cannabis use and the use of social media for information exchange and research. Our analysis reveals the state of the current research and suggests future directions for social media research into cannabis as medicine, particularly the need to identify appropriate data sources for study objectives.

#### Implications for Health Organizations and Regulators

While formal information from health care providers and regulators should be the mainstay of evidence-based information about new therapeutics, the increasing use of social media and other web-based search queries for health advice has emerged as another information source. However, information on the web is not subject to the rigorous standards to which official bodies must adhere. This highlights an important challenge that today’s health care industry faces— offering timely, easily accessible, factual, and practical cannabis information that engages consumers to the same degree as that obtained from web-based forums of like-minded communities and social media.

The fact that consumers obtain experience-based information from fellow users about cannabis consumption indicates that there is a need not only for practical advice but also for much closer scrutiny and regulation of the products they are purchasing, including accurate labeling of the levels of cannabinoids, appropriate doses, and potential side effects.

#### Implications for Clinicians

One of the points raised by the studies is the need to connect patients with more credible and authoritative sources of information, including health practitioners. Other studies have shown that cannabis is not a part of regular medical care, as primary health care providers are either unable to advise on medical cannabis or unwilling to prescribe it [81]. Even for conditions where cannabis is prescribed, it is often only used as an adjunctive treatment or after all regular interventions have failed [82,83].

Research into health care practitioners’ attitudes toward medicinal cannabis has confirmed that even when they support its use, they lack confidence in their knowledge of its benefits, which is tempered by concerns about its risks [84] and legality [85].

Primary providers need greater awareness of the current clinical data and applicability of medicinal cannabis so that they can effectively incorporate cannabis into their treatment plans and properly advise their patients [84]. Currently, instead of learning about medicinal cannabis through structured training programs, physicians are obtaining fragmented knowledge about cannabis from fellow physicians, medical journals, news media, and even from their own patients [86].

## Appendix

Multimedia Appendix 1 Search keywords and inclusion and exclusion criteria.

Multimedia Appendix 2 List of included articles and their analysis.

## Abbreviations

ADHD: attention-deficit/hyperactivity disorder
CAM: complementary and alternative medicine
CBD: cannabidiol
FAERS: Food and Drug Administration’s Adverse Drug Reporting System
PRISMA: Preferred Reporting Items for Systematic Reviews and Meta-Analyses
PTSD: posttraumatic stress disorder
