# Supplementary material for: Using Social Media Data to Investigate Public Perceptions of Cannabis as a Medicine: Narrative Review
Source: J Med Internet Res. 2023 Feb 27;25:e36667. doi: 10.2196/36667 (PMC10012004; doi:10.2196/36667)
Supplement: Multimedia Appendix 1 [file jmir_v25i1e36667_app1.docx]

# Multimedia Appendix 1

Search keywords; inclusion and exclusion criteria

Table 1. Literature database query terms used for finding relevant papers

| **CATEGORY 1 - SOCIAL MEDIA, CANNABIS, AND MEDICAL TERMS** | | |
| --- | --- | --- |
| **Social media related Keywords** | **Cannabis keywords** | **Medical keywords** |
| ‘Social media’ OR Twitter OR reddit OR instagram OR youtube OR pinterest OR facebook OR ‘social network forum’ OR ‘Online health community’ OR ‘message board’ | cannabis OR marijuana OR cannabinoids OR delta-9-tetrahydrocannabinol OR cannabidiol OR cbd OR cbg OR cbn OR thc OR weed | medical OR medicinal OR patient OR patients OR medicine OR doctor OR position OR care OR therapy OR therapeutic |
| **CATEGORY 2 - SOCIAL MEDIA, CANNABIS, AND PSYCHIATRIC DISORDERS** | | |
| **Social media related Keywords** | **Cannabis keywords** | **Psychiatric disorders** |
| ‘Social media’ OR Twitter OR reddit OR instagram OR youtube OR pinterest OR facebook OR ‘social network forum’ OR ‘Online health community’ OR ‘message board’ | cannabis OR marijuana OR cannabinoids OR delta-9-tetrahydrocannabinol OR cannabidiol OR cbd OR cbg OR cbn OR thc OR weed | depression OR depressive OR ‘mental illness*’ OR ‘mental disorder*’ OR ‘mental health’ OR ‘mood disorder*’ OR ‘affective disorder*’ OR anxi* OR ‘panic disorder’ OR ‘obsessive compulsive’ OR adhd OR ‘attention deficit’ OR phobi* OR bipolar OR psychiat* OR psychological OR psychosis OR psychotic OR schizophr* OR ‘severe mental*’ OR ‘serious mental*’ OR antidepress* OR antipsychotic* OR ‘post traumatic*’ OR ‘personality disorder*’ OR stress |
| **CATEGORY 3 - SOCIAL MEDIA, CANNABIS, AND MEDICAL (NON-PSYCHIATRIC) CONDITIONS** | | |
| **Social media related Keywords** | **Cannabis keywords** | **Medical conditions** |
| ‘Social media’ OR Twitter OR reddit OR instagram OR youtube OR pinterest OR facebook OR ‘social network forum’ OR ‘Online health community’ OR ‘message board’ | cannabis OR marijuana OR cannabinoids OR delta-9-tetrahydrocannabinol OR cannabidiol OR cbd OR cbg OR cbn OR thc OR weed | Pain, Opioid, Alzheimer, sleep OR insomnia, inflammatory, arthritis, Multiple Sclerosis, Endometriosis |
| **CATEGORY 4 - SEARCH ENGINE QUERIES AND CANNABIS KEYWORDS** | | |
| **Search Engine keywords** | **Cannabis keywords** | |
| ‘Search engine’ OR ‘search log’ OR ‘search queries’ OR ‘online search’ OR ‘internet Search’ OR ‘web search’ | cannabis OR marijuana OR cannabinoids OR delta-9-tetrahydrocannabinol OR cannabidiol OR CBD OR CBG OR CBN OR thc OR weed | |

Table 2. Inclusion and exclusion criteria

| **Criteria** | **Included** | **Excluded** |
| --- | --- | --- |
| Study type | Primary research studies. | Editorials, letters, commentaries, book chapters, studies where the full text of the publication was not available |
| Intervention | Online user-generated text as a data source. | social media used for recruiting participants.  bots or autonomous systems used as the main data source. |
| Outcome | Studies that were either focused on cannabis and cannabis products that have an impact on health or were health-related, and studies that found medical use of cannabis. | Studies focused on electronic nicotine delivery systems adapted to deliver cannabinoids, and studies focused exclusively on synthetic cannabis. |
| Language | English | Non-English |
| Timeframe | Jan 1974-April 2022 |  |
